# Apitherapy combination improvement of blood pressure, cardiovascular protection, and antioxidant and anti-inflammatory responses in dexamethasone model hypertensive rats

**DOI:** 10.1038/s41598-022-24727-z

**Published:** 2022-12-01

**Authors:** Fatma El-Zahraa Abd El-Hakam, Gomaa Abo Laban, Sahar Badr El-Din, Hala Abd El-Hamid, Mohammed Hamdy Farouk

**Affiliations:** 1 Pharmacology Department, Faculty of Medicine for Girls, Al-Azhar University, Nasr City, 11884 Cairo Egypt; 2 Plant Protection Department, Faculty of Agriculture, Al-Azhar University, Nasr City, 11884 Cairo Egypt; 3 Pathology Department, Faculty of Medicine for Girls, Al-Azhar University, Nasr City, 11884 Cairo Egypt; 4 Animal Production Department, Faculty of Agriculture, Al-Azhar University, Nasr City, 11884 Cairo Egypt

**Keywords:** Drug discovery, Cardiology, Diseases, Medical research

## Abstract

Hypertension-induced ventricular and vascular remodeling causes myocardial infarction, heart failure, and sudden death. Most available pharmaceutical products used to treat hypertension lead to adverse effects on human health. Limited data is available on apitherapy (bee products) combinations for treatment of hypertension. This study aims to evaluate the antihypertensive effects of combinations of natural apitherapy compounds used in the medical sector to treat a variety of diseases. Rats were assigned into six groups consisting of one control group and five hypertensive groups where hypertension (blood pressure > 140/90) was induced with dexamethasone. One of these groups was used as a hypertension model, while the remaining four hypertensive groups were treated with a propolis, royal jelly, and bee venom combination (PRV) at daily oral doses of 0.5, 1.0, and 2.0 mg/kg, and with losartan 10 mg/kg. The PRV combination at all doses decreased arterial blood pressure below the suboptimal value (*p* < 0.001), and PRV combination treatment improved dexamethasone-induced-ECG changes. The same treatment decreased angiotensin-II, endothelin-1, and tumor growth factor β serum levels in hypertensive rats. Additionally, PRV combination improved histopathological structure, and decreased serum levels of NF-kB and oxidative stress biomarkers. We concluded that PRV combination therapy may be used as a potential treatment for a variety of cardiovascular diseases.

## Introduction

Hypertension is a growing disorder and represents a leading indicator for risk for all cardiovascular diseases and mortality^1^. For instance, hypertension-induced left ventricular remodeling causes heart failure, coronary artery diseases, and eventual death^2^. Approximately 1.4 billion people worldwide have suffered from hypertension and its complications. Approximately 31.1% of people suffer from primary or secondary hypertension^3^. Some medications, such as glucocorticoids which are widely used to treat unrelated medical conditions, may cause iatrogenic hypertension and lead to adverse effects^4^. Furthermore, long-term glucocorticoid treatment causes hypertension in 80–90% of patients^5^. Dexamethasone (Dex) is the strongest synthetic glucocorticoid, with pure glucocorticoid activity, compared with natural cortisol and corticosterone. These synthetic compounds possess anti-inflammatory and immunosuppressive effects^6^.

Honeybee products possess numerous beneficial pharmacological and biological properties, and interestingly, these properties have been widely used in folk medicines^7^. Apitherapy is a medical term that refers to the therapeutic use of honeybee-related ingredients and their derivatives, including royal jelly, propolis, bee pollen, bee venom, beeswax, etc^8^. For instance, apitherapy possesses antioxidant and free-radical scavenging properties. Additionally, such therapy contains various classes of phytochemicals such as flavonoids, aromatic acids, and phenolic elements. Although previous experiments have had very positive results when examining the cardiovascular impacts of honeybee products, the effects of apitherapy combinations still remains mostly ignored^9^.

Honey has had an ethno-pharmacological relevance since the dawn of civilization. For instance, ancient Egyptians, Chinese, and Greeks recommended honey for treating various disorders, including healing of wounds and various digestive diseases. They also recommended using honey and vinegar together to relieve discomfort. Furthermore, religious books, including the Veda, Bible, and Qur'an emphasize the advantages of honey^10^. Many medical disorders call for the use of honey as a wound healer, anti-inflammatory, anti-diabetic, etc. This food provides different health advantages and possesses a low glycemic index, and today honey is preferred and more popular than other sugars^11^. Several ancient luminaries, including Aristotle, Aristoxenus, Porphyry, Cornelius Celsus, and Dioscorides recommended various types of honey to treat diverse ailments^12^.

There is a bidirectional link between oxidative stress (OS) and the incidence of hypertension^13–15^. Apitherapeutic substances control cardiovascular system functions by inhibiting the OS that causes hypertension. Such substances support the development of the antioxidant system, lower blood pressure, and halt initiation and progression of diseases brought on by hypertension ^16^.

Propolis, bee venom, and royal jelly (RJ) are quite well known health foods^17^. Propolis is a phenolic compound manufactured by bees from plant resins^18^. This phenolic compound can be used as an antimicrobial, antioxidative, anti-ulcer, and anti-tumor agent. Propolis is also used in aesthetics. Additionally, blood pressure and cholesterol levels are reduced through use of propolis^18^. Propolis contains different flavonoid component such as caffeic acid, *p*-Coumaric acid, and ferulic acid. Furthermore, propolis contains phenolic compounds, wax, dry residue, and volatile substances^19^. Similarly, RJ contains a variety of bioactive molecules, possessing antibacterial, antioxidant, anti-inflammatory, immunomodulatory, anticancer, antihypertensive, and vasodilatory properties^20^. RJ contains 60–70% water, 12–15% crude protein, 10–16% total sugar, and 3–6% lipids, vitamins, salts and free amino acid^21^. Bees use their venom to protect their hives. Their venom contains bioactive chemicals such melittin, apamine, phospholipase 2, histamine, dopamine, norepinephrine, and others substances^22^.

Bee venom therapy has been used as an alternative and complementary therapy. Research has indicated that bee venom used therapeutically possesses anti-inflammatory, anti-apoptosis, anti-fibrosis, and anti-arthrosclerosis characteristics^23,24^. Although apitherapy is used in several regions of the world to improve health, available apitherapy combinations are limited^22^. Numerous synthetic medications are used to control hypertension, although the administration of synthetic antihypertensive medications has several harmful side effects. Cough, edema, flushing, headache, excessive urination (polyuria), arrhythmias, wheezing/dyspnea, and dizziness are examples of side effects that can be severe enough to prevent adherence to antihypertensive medicine regimens^25^. The long-term safety of some antihypertensive agents has become a concern since their use may have lethal effects, e.g., calcium channel blockers, beta blockers, and diuretics increase breast cancer risk. Additionally, diuretics may elevate the risk of breast cancer-specific mortality as reported by Xie et al.^26^. Thus, there is a growing interest in developing natural antihypertensive compounds to reduce blood pressure and its consequences^27^. According to Zambrano et al.^28^, and Tanuğur Samancı and Kekeçoğlu^29^, using bee product combinations instead of conventional therapy may have favorable effects. Zambrano et al.^28^ has proven apitherapy combination efficacy in COVID-19. Also, Tanuğur Samancı and Kekeçoğlu^29^ identified similar combination efficacy as a dermatological preparation. Additionally, Kas et al.^30^ demonstrated the effectiveness of various bee products (honey, pollen, and bee bread) as treatments for patients with atherogenic dyslipidemia in a trial which included 157 patients. Furthermore, Andriţoiu et al.^31^ showed that apitherapy diet formulations mitigated liver, spleen, and pancreas toxicity induced by carbon tetrachloride in rats following 9 weeks of treatment.

Cardiometabolic diseases such as metabolic syndrome and obesity were also clinically improved (in the form of reductions of overweight, relative body mass, glycemia, high triglycerides level, as well as systolic and diastolic blood pressures) after treatment with an apitherapy combination containing propolis, bee pollen, RJ, and multi-flower honey. An apitherapy combination (propolis, bee pollen, and RJ) was used in a clinical study involving 68 patients where metabolic and cardiovascular system parameters were improved significantly. These parameters included body mass, lean body mass, fat tissue mass, serum lipids, blood sugar, uric acid, arterial pressure, and pulse rate before and after functional loading tests^32^. Furthermore, a combination of propolis and bee venom showed a marked curative effect on OS produced by gamma radiation in rats^33^. Additionally, a combination treatment consisting of aqueous extract of propolis and bee venom induced synergistic antiproliferative effects in breast cancer cells^34^. Other synergistic effects have been investigated involving using mixtures of honey, propolis, pollen, and RJ to mitigate the toxic effects of insecticides in rats^35^. An earlier study assumed that antioxidant and anti-inflammatory mechanisms were responsible for the effectiveness of the apitherapy mixture. Although consumers can select from a variety of bee products, the optimal combination of these products is not well characterized. Therefore, it has been hypothesized that apitherapy combinations can potentiate their constituent ingredients and may provide more favorable therapeutic effects in hypertension treatment than does a solitary product.

The present study aimed to evaluate the antihypertensive effects of an apitherapy combination based on natural and widely used compounds in the medical sector to treat a variety of diseases. The resulting insights may offer novel, risk-free treatments for hypertension and its sequelae such as tissue remodeling.

## Results

Table 1 and Figure S1 demonstrate that higher systolic blood pressure (SBP) was seen in the model group (*p* < 0.001) compared with the control group, while lower SBP was observed in the apitherapy compound (PRV) 2 mg/kg group. Similar trends were observed in diastolic blood pressure and mean arterial blood pressure.

**Table 1:** Mean ± SD for the arterial blood pressure measurements among different experimental groups.

Item	Experimental groups						F value	*p*-value
	Control (n = 6)	Model (n = 6)	PRV 0.5 mg/kg (n = 6)	PRV1 mg/kg (n = 6)	PRV2 mg/kg (n = 6)	Losartan (n = 6)		
Systolic blood pressure (mmHg)	89.53 ± 6.08 a	148.75 ± 10.12a	121.67 ± 4.38ab	97.00 ± 3.07bc	95.47 ± 6.77bc	98.47 ± 2.19bc	84.150	< 0.001
Diastolic blood pressure (mmHg)	57.66 ± 4.72 a	105.18 ± 6.68a	97.79 ± 2.32a	60.09 ± 2.26bc	58.66 ± 7.06bc	71.80 ± 4.71abcde	107.414	< 0.001
Mean arterial blood pressure (mmHg)	68.27 ± 4.46 a	119.70 ± 7.51a	111.01 ± 2.99ab	77.50 ± 2.83abc	70.93 ± 5.10bc	85.10 ± 2.92abce	131.252	< 0.001

The results are presented as the means ± standard deviation (SD). The groups with different superscripts in the same row represent a statistical significance (*p* < 0.05), a: control; b: model (dexamethasone-treated group); c: PRV 0.5 mg/kg; d: PRV1mg/kg; e: PRV 2 mg/kg.

*n*  number/group, *PRV* propolis, royal jelly & bee venom.

In terms of ECG, Dex caused a considerable (*p* < 0.001) decrease in heart rate (bradycardia) associated with extended PR and QRS periods, as well as increased frequency of ventricular tachycardia and falling beats, compared with the control group. Using a dose–response approach, the PRV significantly normalized ECG results, increased heart rate, and shortened both PR and QRS intervals. Additionally, ventricular tachycardia and falling beats were considerably reduced in all PVR treated groups compared with the model group. Losartan also significantly improved ECG results and was comparable to PRV in all ECG measured parameters as shown in Table 2.

**Table 2:** Mean ± SD for electrocardiogram (ECG) records among different experimental groups**.**

Item	Experimental groups						F value	*p*-value
	Control (n = 6)	Model (n = 6)	PRV 0.5 mg/kg (n = 6)	PRV1 mg/kg (n = 6)	PRV2 mg/kg (n = 6)	Losartan (n = 6)		
HR (beat/min)	296.00 ± 45.31 a	124.63 ± 30.94a	197.35 ± 20.15ab	284.73 ± 47.81bc	306.33 ± 20.93bc	306.47 ± 39.83bc	26.222	< 0.001
PR (pbm)	33.57 ± 2.86 a	52.94 ± 4.25a	48.10 ± 5.84a	33.42 ± 2.43bc	33.16 ± 4.29bc	36.11 ± 3.86bc	26.319	< 0.001
QRS (ms)	10.67 ± 0.99 a	84.33 ± 20.23a	76.00 ± 22.36a	12.16 ± 1.34bc	10.69 ± 1.62bc	12.81 ± 1.11bc	49.613	< 0.001

The results are presented as the means ± standard deviation (SD). The groups with different superscripts in the same row represent a statistical significance (*p* < 0.05), a: control; b: model (dexamethasone-treated group); c: PRV 0.5 mg/kg; d: PRV1mg/kg; e: PRV 2 mg/kg.

*n* number /group, *HR* heart rate, *PRV* propolis, royal jelly & bee venom.

Table 3 shows the results of serum level hypertension indices (Ang II, TGF-β, and ET-1). The Dex treatment in the model group had higher levels of all hypertension indices including Ang II, TGF-β, and ET-1 levels compared with the control group.

**Table 3:** Mean ± SD for biochemical analysis of serum hypertension indexes in different experiemntal groups.

Items	Experimental groups						F value	*p*-value
	Control (n = 6)	Model (n = 6)	PRV 0.5 mg/kg (n = 6)	PRV1 mg/kg (n = 6)	PRV2 mg/kg (n = 6)	Losartan (n = 6)		
Angiotensin II (ng/mL)	23.18 ± 2.57 a	95.88 ± 10.00a	62.85 ± 9.35ab	46.98 ± 23.54ab	42.17 ± 7.33abcd	40.67 ± 7.51abcd	65.718	< 0.001
TGF-β (pg/mL)	54.52 ± 6.91 a	121.62 ± 5.07a	69.03 ± 6.41ab	67.27 ± 6.58ab	53.52 ± 4.96bcd	72.93 ± 9.02abe	85.546	< 0.001
Endothlin-1 (ng/mL)	4.18 ± 0.48 a	16.07 ± 2.89a	9.27 ± 1.06ab	6.18 ± 1.01bc	4.93 ± 0.48bc	4.82 ± 0.75bc	64.234	< 0.001

The results are presented as the means ± standard deviation (SD). The groups with different superscripts in the same row are significantly different (*p* < 0.05), a: control group; b: model (dexamethasone-treated) group; c: PRV 0.5 mg/kg group; d: PRV1mg/kg group; e: PRV 2 mg/kg group.

*n* number/group, *PRV* propolis, royal jelly & bee venom, *TGF-β *transforming growth factor beta.

Treatment with PRV resulted in dose-dependent reductions in Ang II, TGF-β, and ET-1 serum levels. The decline in ET-1 serum level was observed in both the 2 mg PRV and losartan-treated groups. Furthermore, using the 2 g/kg PRV dose markedly decreased TGF-β serum levels compared with lower PRV doses and was comparable to the control group. A considerable decrease was shown in TGF-β levels with losartan treatment compared with the control group, but 2 gm/kg PRV treatment had the lowest TGF-β level compared to the other groups.

Results of serum OS biomarkers and nuclear factor kappa-light-chain-enhancer of activated B cells (NF-κB) are shown in Table 4. Findings confirmed that Dex treatment in the model group had higher levels of MDA and NF-κB, and lower SOD, CAT, and GSH serum levels compared with the control group (*p* < 0.001). Opposite trends were noticed in all doses of PRV treatments (0.5, 1.0, and 2 mg/kg), in a dose-dependent manner. Generally, 2 mg/kg PRV treatment improved OS and NF-κB inflammatory marker levels compared with the other PRV treatments. The 2 mg PRV and losartan treatments had significantly lower MDA levels compared with the control group. Additionally, losartan treatment had a higher SOD serum level, with an effect comparable to all PRV treatments. The levels of GSH were increased in PRV treatments in a dose-dependent manner. Losartan had an effect equivalent to the 1 mg PRV treatment.

**Table 4:** Mean ± SD for biochemical analysis of serum oxidative stress biomarkers and NF-κB in different experimental groups.

Item	Experimental groups						F value	*p*-value
	Control (n = 6)	Model (n = 6)	PRV 0.5 mg/kg (n = 6)	PRV1 mg/kg (n = 6)	PRV2 mg/kg (n = 6)	Losartan (n = 6)		
MDA (µmol/mL)	13.48 ± 0.96 a	108.77 ± 8.76a	47.02 ± 6.41ab	44.18 ± 5.82ab	33.37 ± 4.67abcd	43.65 ± 3.13ab	200.520	< 0.001
SOD (IU/mL)	23.83 ± 3.22 a	9.20 ± 1.06a	20.33 ± 1.18b	19.20 ± 2.50ab	19.10 ± 2.08abc	20.53 ± 1.95b	32.522	< 0.001
Catalase (µmol/L)	120.60 ± 5.18 a	55.60 ± 8.22a	95.12 ± 6.58b	98.35 ± 9.08b	153.75 ± 39.46abcd	100.12 ± 6.10be	20.710	< 0.001
GSH (µmol/mL)	62.93 ± 3.41 a	27.72 ± 3.59a	48.45 ± 3.49ab	56.08 ± 2.69b	56.63 ± 7.91bc	46.27 ± 3.32abde	46.614	< 0.001
NF-κB (ng/mL)	111.50 ± 5.84 a	282.80 ± 35.88a	175.98 ± 4.98ab	146.00 ± 7.45abc	128.73 ± 6.09bc	147.17 ± 8.02abc	90.307	< 0.001

The results are presented as the means ± standard deviation (SD). The groups with different superscripts in the same row represent a statistical significance (*p* < 0.05), a: control group; b: model (dexamethasone-treated) group; c: PRV 0.5 mg/kg group; d: PRV1mg/kg group; e: PRV 2 mg/kg group.

*n*  number/group, *PRV* propolis, royal jelly & bee venom, *MDA* malonaldehyde, *SOD *superoxide dismutase, *GSH* reduced glutathione, *NF-κB* nuclear factor kappa-light-chain-enhancer of activated B cells.

**Figure 1:**
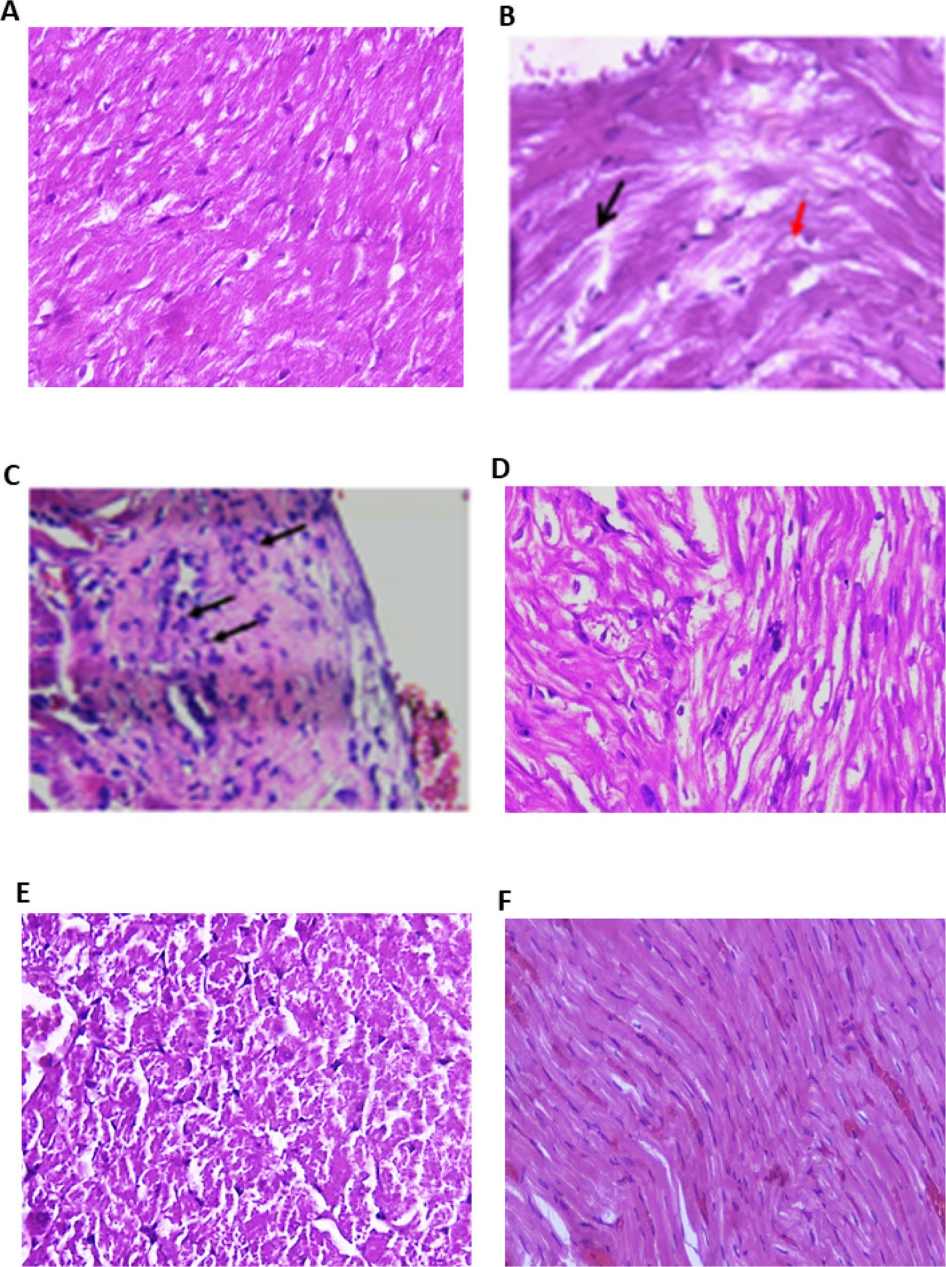
Photomicrograph of heart tissue sections in different studied groups. (H&E). (**A**) Control group showed normal myofibrillar structure with striations, ×200; (**B**) model group (dexamethasone-treated group) showed muscle separation (black arrow) with some vacuolated cells (red arrow) to myositis with scattered inflammatory cells (arrows) surrounding dilated blood vessels with stromal edema (×400); (**C**) PRV 0.5 mg/kg group showed slight separation of the cardiac muscle (black arrow), vacuolization of some cells (red arrow), and scattered few inflammatory cells; (**D**) PRV 1 mg/kg group showed greater improvement of the histopathological picture, except for muscle separation; (**E**) PRV 2 mg/kg group showed congested myocardium reverted to a nearly normal histopathological appearance; (**F**) losartan-treated group showed partial loss of cardiac muscle striations and occasional muscle separation (×200).

The hematoxylin and eosin (H&E stain) data used for cardiac tissue examination is shown in Fig. 1. Sections of cardiac tissue from normal control rats revealed myocytes with intact cell membranes, a normal myofibrillar structure with striations, and the appearance of branched and continuous myofibrils. There was no evidence of infiltration of inflammatory cells, edema, inflammation, or necrosis (Fig. 1A). Cardiac tissues from model rats treated with Dex exhibited a wide variety of pathological features, ranging from muscle separation (black arrow) with apparent vacuolated cells (red arrow; Fig. 1B), to myositis with scattered inflammatory cells (black arrows) surrounding dilated blood vessels with stromal edema (Fig. 1B). Compared with the model group, PRV treatment significantly improved myocardial affection. The 0.5 mg PRV treatment sample showed a slight separation of the cardiac muscle (black arrow), vacuolization of cells (red arrow), and a few scattered inflammatory cells, as depicted in Fig. 1C. Treatment with 1 mg PRV showed greater histopathological improvement, except for muscle separation (Fig. 1D). However, the 2 mg PRV treatment showed that a congested myocardium reverted to a nearly normal histopathological appearance, as shown in Fig. 1E. Myocardial fibers were closely packed in the losartan-treated group, indicating a partial loss of cardiac muscle striations and occasional muscle separation (Fig. 1F).

**Figure 2:**
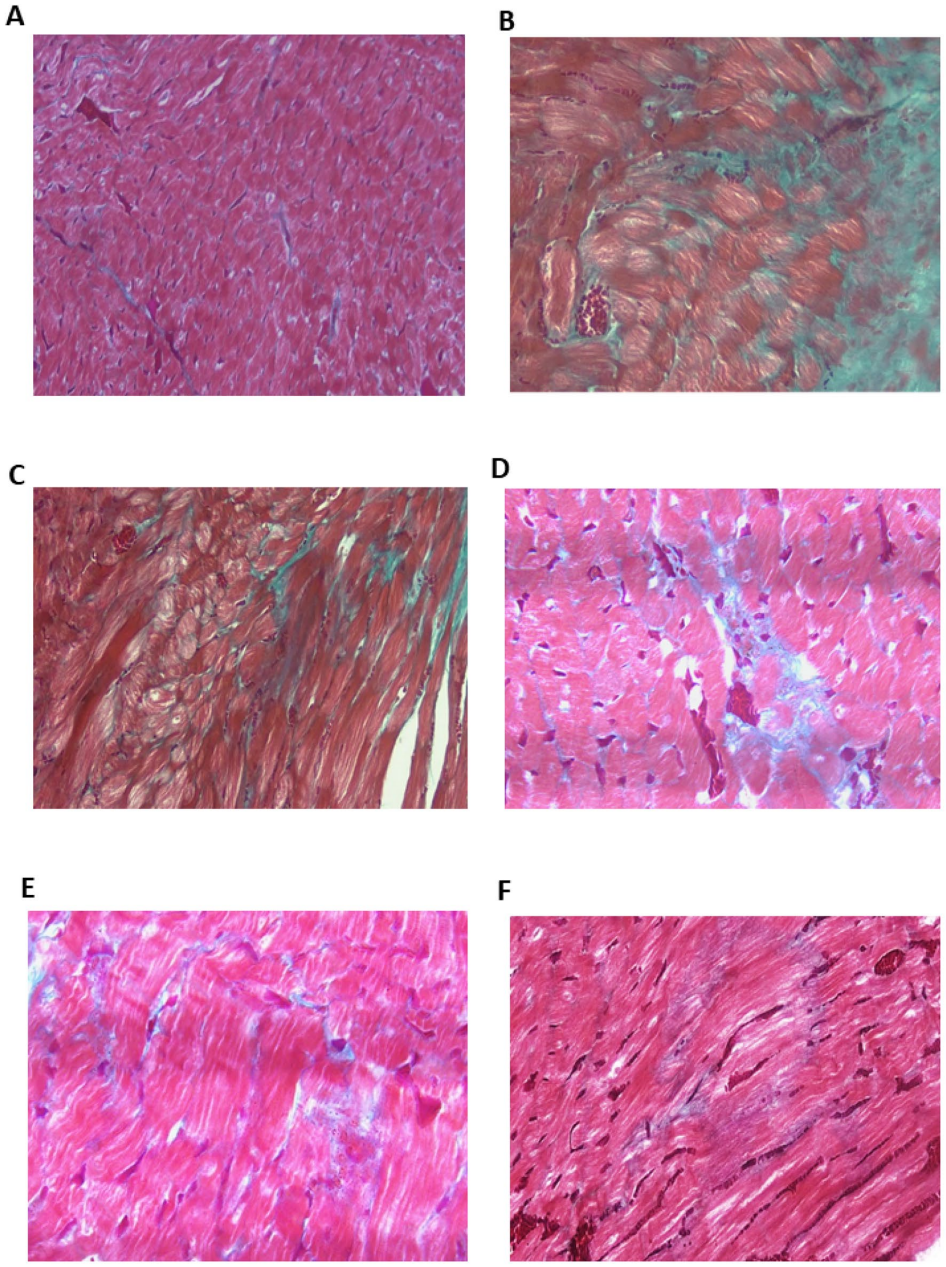
Photomicrograph of heart tissue sections in different studied groups using Masson’s trichrome staining. (**A**) Control group exhibited a minimal amount of collagen fiber deposition around blood vessels (×200); (**B**) model group (dexamethasone-treated group) showed prominent and dense collagen fibrosis in the left ventricle (×400); fibrosis occurred in both interstitial and perivascular regions in an unorganized manner; (**C**) PRV 0.5 mg/kg; (**D**) PRV 1 mg/kg; (**E**) PRV 2 mg/kg with less fibrosis (×400). (**F**) Losartan treatment reduces cardiac fibrosis and collagen fibers are deposited around blood vessels (arrows) (×400).

**Figure 3:**
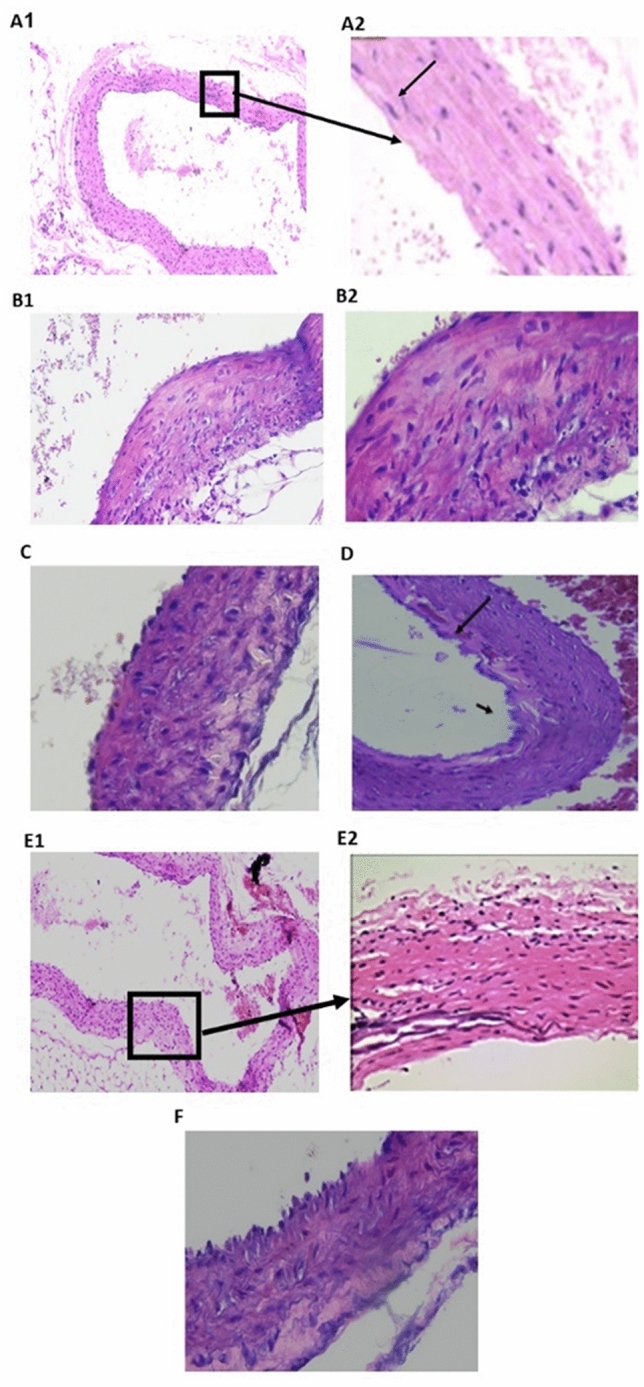
Photomicrograph of aorta tissue sections in different studied groups. (H&E) (**A**) Control group: the tunica intima (arrowhead) faces the lumen. The tunica media is composed of elastic fibers and smooth muscle (thick arrow). The tunica adventitia is the outermost layer, which is composed of loose connective tissue (arrow) (**A1,A2**, respectively; ×100, ×200); **(B1,B2**) model group (dexamethasone- treated group) exhibited pathological changes, including lumen bulging and medial inflammatory cell infiltration (arrow), as well as an increase in the tunica media thickness overall (**B1**, ×200  and **B2**, ×400); (**C**) PRV 0.5 mg/kg there was a decrease in wall thickness associated with crystalloid material deposition (arrow); (**D**) PRV 1 mg/kg showed a decrease in wall thickness with organization of muscular fibers, with focal areas of disrupted endothelial cell lining fibrillation and focal subintimal hemorrhage (×200); (**E**) PRV 2 mg/kg with nearly normal endothelial lining and normal muscle structure (×200); (**F**) losartan-treated group with reduced intimal thickness.

Data regarding Masson’s trichrome staining used for examining cardiac tissue is depicted in Fig. 3. Masson's trichrome staining is a widely used technique for detecting collagen fibers and myocardial fibrosis. As indicated by the bright blue staining, the control group exhibited a minimal amount of collagen fiber deposition around blood vessels (Fig. 2A). Prominent and dense collagen fibrosis was observed in the left ventricle of the Dex-induced hypertensive rats (Fig. 2B), and unorganized fibrosis occurred in both interstitial and perivascular regions. As shown in Fig. 2C–E, all PRV treatments showed less fibrosis in a dose-dependent manner. Additionally, the losartan treatment showed less cardiac fibrosis and collagen fibers deposited around blood vessels (arrows) as depicted in Fig. 2F.

The aortic tissue examination (H&E stain) is depicted in Fig. 3. A photomicrograph of a thoracic aorta section from the control group indicated that the tunica intima (arrowhead) faced the lumen. The tunica media was composed of elastic fibers and smooth muscle (thick arrow). The tunica adventitia was the outermost layer (Fig. 3A1), which was composed of loose connective tissue (arrow) (Fig. 3A2). The aortic tissues of the hypertensive rats in the model group exhibited pathological changes, including lumen bulging and medial inflammatory cell infiltration (arrow), as well as an increase in the overall tunica media thickness (Fig. 3B1,B2). PRV treatments showed a gradual dose-dependent improvement to aortic tissue (Fig. 3C). The 0.5 mg PRV treatment showed a decrease in wall thickness associated with crystalloid material deposition (arrow). The 1.0 mg PRV treatment showed a greater improvement compared with the control group and a decrease in wall thickness associated with organization of muscular fibers, as well as the appearance of focal areas of disrupted endothelial cell lining fibrillation and focal subintimal hemorrhage (Fig. 3D). The 2 mg PRV treatment showed nearly normal endothelial lining and normal muscle structure (Fig. 3E1,E2), and the losartan treatment showed thinner intimal thickness than seen with the 2 mg PRV treatment (Fig. 3F).

**Figure 4:**
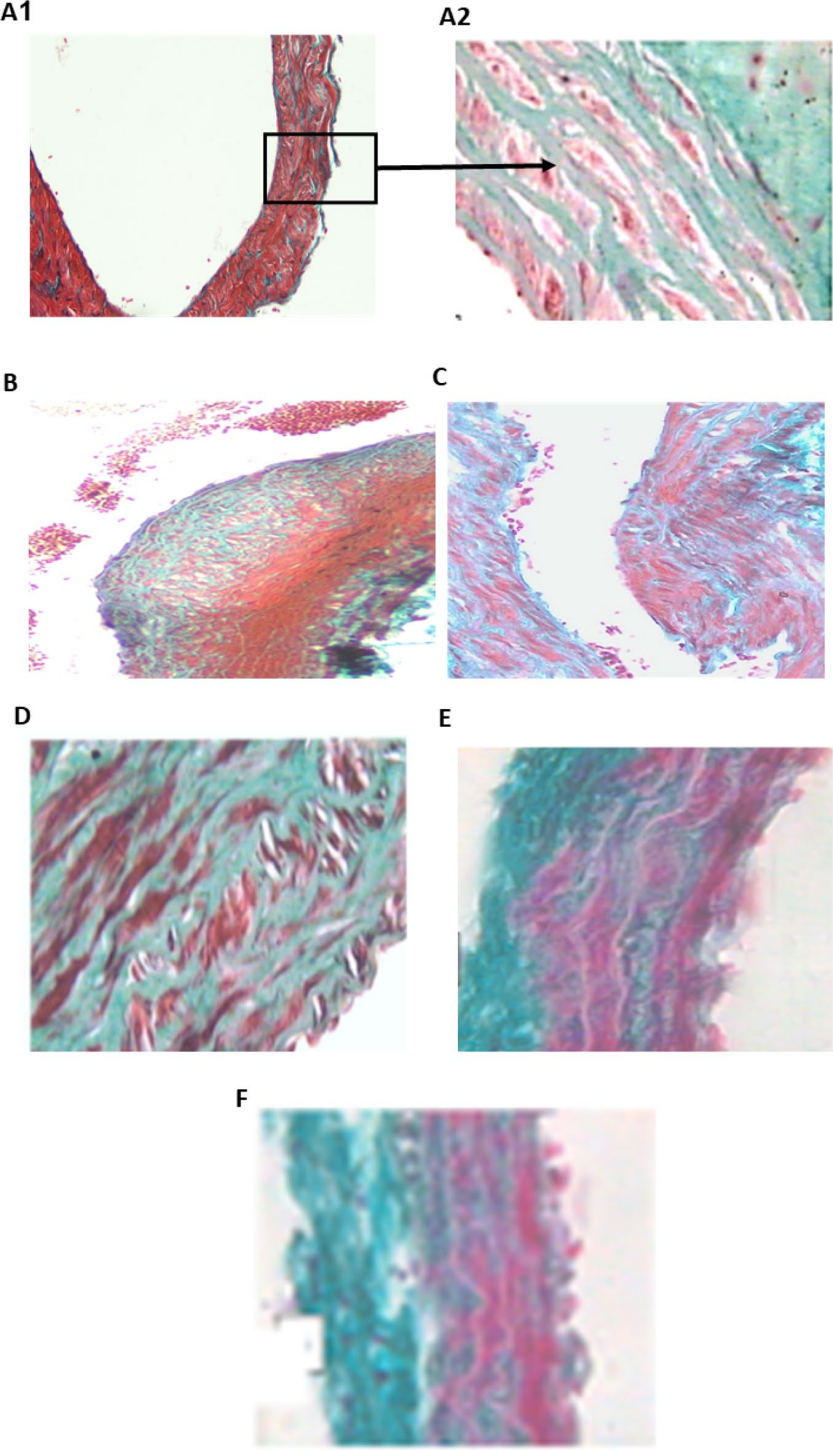
Photomicrograph of thoracic aorta sections in different studied groups using Masson’s trichrome staining. (**A**) Control group tunica media, demonstrated wavy elastic collagen fibers with smooth muscle cells interspersed (**A1**, ×100, **A2**, ×400); (**B**) model group (dexamethasone-treated group) showed an unorganized accumulation of collagen fibers with increase in smooth muscle cell layers in the subintima, and endothelium bulging and tearing (×200); (**C**) PRV 0.5 mg/kg; (**D**) PRV 1 mg/kg; (**E**) PRV 2 mg/kg; (**F**) losartan-treated group. The last 4 groups showed tunica media as wavy elastic collagen fibers and smooth muscle cells associated with reversal of dense fibrosis and the re-establishment of intimal integrity (**C**, ×200, **D–F** ×400).

**Figure 5:**
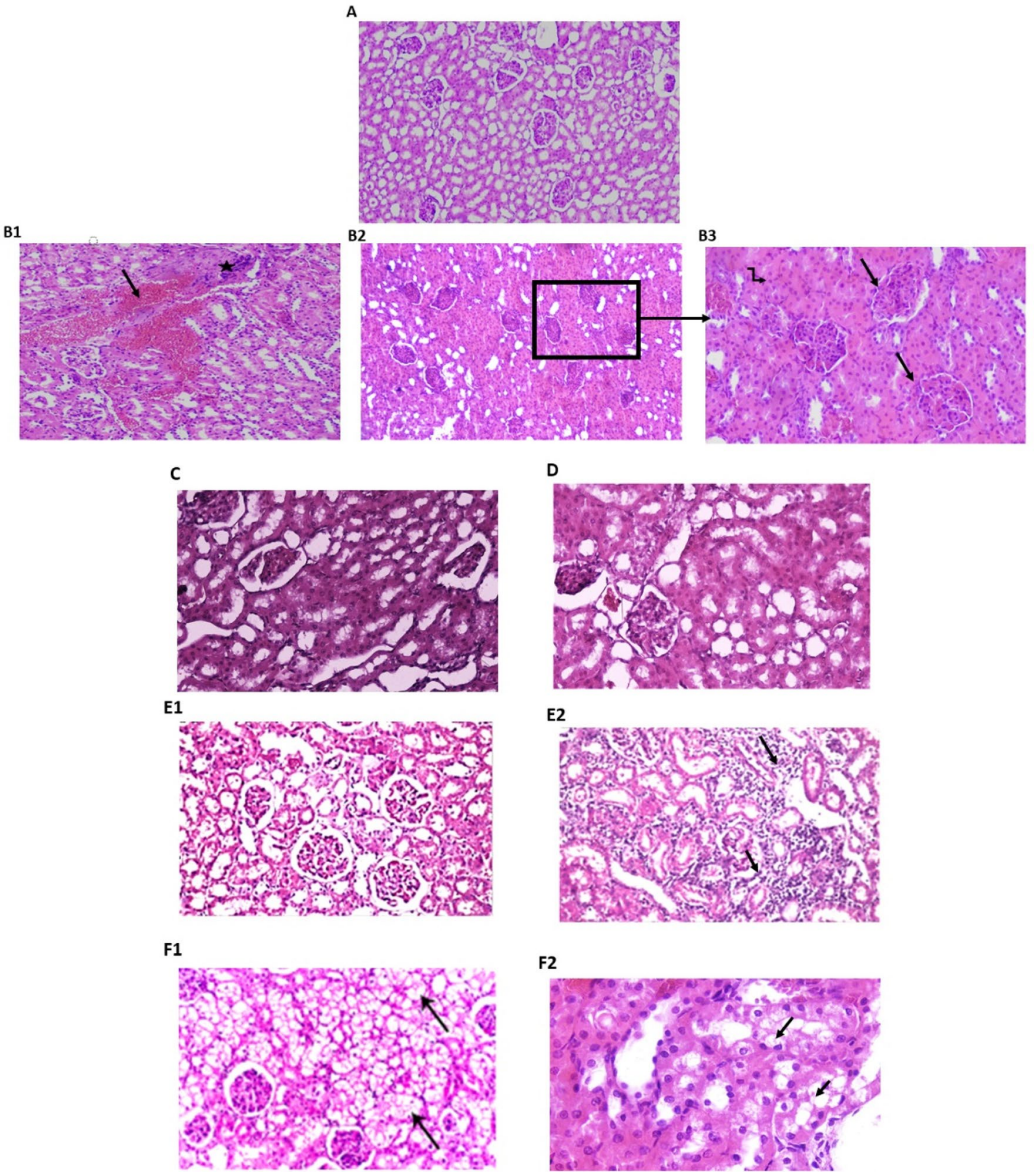
Photomicrograph of kidney tissue sections in different studied groups. (H&E) (**A**) control group with normal histological structure of glomeruli and tubules (H&E ×200); (**B1,B2**) model group (dexamethasone-treated group) interstitial hemorrhage (arrow) ×100, hypercellular glomeruli (arrow) in (**B3**), ×400, obliterated tubules by swollen epithelial cells (zigzag arrow in **B3**); (**C**) PRV 0.5 mg/kg showing focally shrunken glomeruli and focally swollen hyalinized epithelial cells lining the tubules; (**D**) PRV1 mg/kg with well-formed glomeruli and tubules; (**E**) PRV 2 mg/kg exhibited a nearly normal histological renal pattern (**E1**, ×200) (arrows) with some inflammatory infiltrations (arrow **E2**); (**F**) losartan-treated group showing improved glomerular picture with interstitial vacuolation (**F1**, arrows), swollen hyalinized epithelial cells lining the tubules (arrows) were observed (**F2**, ×200).

Data of Masson’s trichrome staining used for aortic tissue examination is shown in Fig. 5. In the tunica media, the control group had wavy elastic collagen fibers with interspersed smooth muscle cells (Fig. 4A1,A2). The Dex treatment sample showed an unorganized accumulation of collagen fibers and an increase in smooth muscle cell layers in the subintima, inducing endothelium bulging and tearing (Fig. 4B). However, PRV treatments showed lower fibrosis in a dose-dependent manner and higher collagen deposition in the aortic wall. In the PRV and losartan-treated groups, the aorta tunica media samples appeared as wavy elastic collagen fibers and smooth muscle cells. The same treated groups had lower aortic fibrosis compared with the model group, with re-established intimal integrity (Fig. 4C–F).

Data of H&E staining used for renal tissue examination is depicted in Fig. 5. Normal histological structure of glomeruli and tubules were observed in the control group (Fig. 5A). Dex treatment in the model group showed a variety of pathological features, ranging from interstitial hemorrhage (Fig. 5B1), to hypercellular glomeruli (Fig. 5B2,B3), and obliterated tubules associated with swollen epithelial cells (zigzag arrow in Fig. 5B3). All PRV treatments showed histopathological deterioration caused by Dex treatment. Kidney sections from the 0.5 mg PRV treatment group indicated focally shrunken glomeruli and focally swollen hyalinized epithelial cells lining the tubules (Fig. 5C). The 1 mg PRV treatment showed well-formed glomeruli and tubules (Fig. 5D), and the 2 mg PRV treatment exhibited a nearly normal histological renal pattern (Fig. 5E1) (arrows) except for inflammatory infiltration (Fig. 5E2). Losartan treatment improved the glomerular picture with interstitial vacuolation (arrows, Fig. 5F1), and swollen hyalinized epithelial cells were observed lining the tubules (arrows, Fig. 5F2).

**Figure 6:**
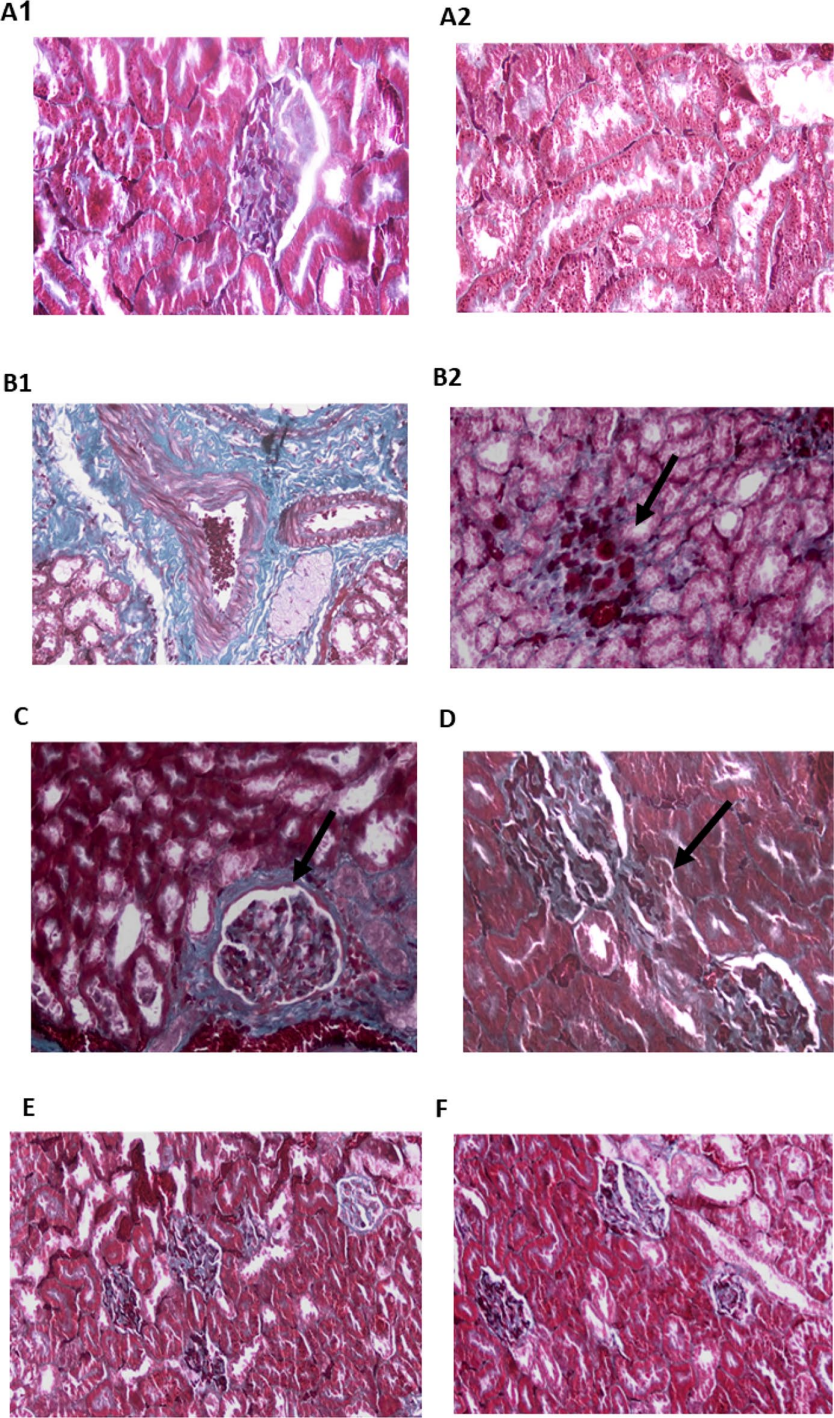
Photomicrograph of kidney tissue sections in different studied groups using Masson’s trichrome staining. (**A**) Control group; (**B1,B2**) model group (dexamethasone-treated group) showing diverse array of pathological features, ranging from stromal interstitial fibrosis surrounding tubules and glomeruli (**B1**, ×400) to fibrotic glomeruli with remnants of glomerular capillaries (arrow) (**B2**; ×400); (**C**) PRV 0.5 mg/kg slight decrease of fibrosis with remnants around the glomeruli remained (arrow); (**D**) PRV 1 mg/kg showing less interstitial fibrosis (arrow) between the glomeruli and a better histopathological pattern (**D**, ×400); (**E**) PRV 2 mg/kg; (**F**) losartan-treated group. Both PRV2 mg/kg and losartan treated groups reversed dexamethasone-induced fibrosis with normal glomeruli and tubules as well as some degree of hypercellular glomeruli and occasional shrinking glomeruli in the losartan-treated group.

Data of Masson’s trichrome staining used for renal tissue examination is depicted in Fig. 6. The control architecture was normal, with few fibrous tissues surrounding tubules (Fig. 6A1,A2) Dex-induced hypertension displayed a diverse array of pathological features, ranging from stromal interstitial fibrosis surrounding tubules and glomeruli (Fig. 6B1) to fibrotic glomeruli with remnants of glomerular capillaries (arrow, Fig. 6B2). Interstitial fibrosis was improved in the 0.5 mg PRV treatment group, but fibrosis around the glomeruli remained unchanged (arrow, Fig. 6C). However, the 1 mg PRV treatment showed less interstitial fibrosis (arrow) between the glomeruli and a better histopathological pattern (Fig. 6D). The sections from 2 mg PRV (Fig. 6E, 200×) and losartan (Fig. 6F) treatments reversed Dex-induced fibrosis and revealed normal glomeruli and tubules with some degree of hypercellular glomeruli and occasional shrinking glomeruli in the losartan-treated group.

## Discussion

Hypertension can initiate and develop ventricular and aortic tissue remodeling and lead to kidney damage. Antihypertensive therapies can lead to unfavorable adverse effects and alter the clinical course of the disease. Therefore, finding natural and safe alternative therapies is a major goal. In the present study, the efficacy of an apitherapy combination in Dex-induced model hypertensive rats was evaluated. Apitherapy combinations were found to have significantly lowered arterial blood pressure and showed cardiovascular and renal protective effects in the hypertensive rats.

In the present study, 10 μg/kg/day^−1^ of Dex treatment for 6 weeks resulted in increased arterial blood pressure. This finding was consistent with Dubey et al.^5^, and Safaeian and Zabolian^36^ who found similar results using the same dosage of Dex and for the same period of treatment in rats. As noted, a 6-week treatment of PRV reduced Dex-induced hypertension (Dex-HT) in a dose-dependent manner. Similarly, losartan-reduced Dex-HT, which concurs with the findings of Azevedo et al. (2003) who found that losartan (10 mg/kg/day^−1^) for 18 weeks lowered blood pressure in spontaneously hypertensive rats. Additionally, Sun et al.^27^ noticed that valsartan (angiotensin II receptor antagonist) improved hypertension in spontenously hypertensive rats after 5 weeks of treatment. Furthermore, Ozdemir et al.^16^ reported that propolis could reduce N^G^-nitro-l-arginine methyl ester (l-NAME) and induces systolic hypertension after 28 days of treatment. The research also revealed that Dex treatment induced ECG changes including bradycardia and ventricular arrhythmia (ventricular premature beats). A similar trend was observed by Macedo et al.^37^ who found that a 7 days 2 mg intraperitoneal Dex treatment could increase blood pressure and was associated with cardiac hypertrophy and arrhythmias. The treatments with 1 mg/kg and 2 mg/kg PRV normalized heart rate and indicated ventricular arrhythmia with significant decreases seen in PR and QRS intervals, compared with the Dex-treated group. Increased arrhythmogenicity and ventricular fibrillation are related to cardiac structural remodeling caused by heart failure^38^. Furthermore, cardiac fibrosis reduces heart rate and causes ventricular arrhythmias by impairing electrical conduction and resulting in the formation of re-entry circuits^39,40^. This could explain the ECG changes that were induced by Dex in the present study.

Cardiotoxicity can be reduced by apitherapeutic agents^16^. Silva et al.^18^ stated that propolis extracts promote antioxidant and anti-inflammatory activity. Malekinejad et al.^41^ proposed that RJ mitigates paclitaxel-induced cardiotoxicity in rats and provides cardioprotective effects in the presence of ischemia-induced cardiac injury. Additionally, Gu et al.^42^ demonstrated that bee venom contains an active ingredient (apamin) which provides a calcium channel blocking effect which leads to increased action potential duration in human heart transplant studies and in animal heart failure models. These were associated with a decreased arrhythmogenic effect and correlated with findings of this study. Additionally, several studies have demonstrated that apamin treatment reduces the duration of ventricular fibrillation^43,44^.

Chronic Dex treatment induces myocyte hypertrophy in rats^45^. The mechanism of cardiac remodeling (hypertrophy/proliferation, fibrosis), hypoxia, and decreased left ventricular function is related to activation of the angiotensin II (Ang II) pathway, (the most potent stimulator of left ventricular remodeling)^46,47^. In cardiomyocytes, Ang II activates G protein-dependent signaling pathways, eliciting Ca^2+^ entry, and initiating cardiomyocyte hypertrophy ^48^. Sustained increases in intracellular Ca^2+^ concentrations ([Ca^2+^]i) induce pathological myocardial hypertrophy via activation of Ca^2+^ dependent signaling pathways (such as Ca^2+^ calcineurin). Such pathways participate in a number of extracellular signaling mechanisms which result in myocardial hypertrophy^49^. Cardiac hypertrophy is a primary adaptive process, leading to chronic hypertrophy causing heart failure, ventricular dilatation, and sudden death^50^. Therefore, in cardiac diseases, Ang II inhibition reduces left ventricular remodeling and improves heart function^51^. Similarly, Ang II can enhance vascular smooth muscle cell (VSMC) growth by inducing mitogen-activated protein kinases during apoptosis or growth. Thus, arterial stiffness occurs in association with increased peripheral resistance which has been shown in hypertension cases^52,53^.

Endothelin-1 (ET-1) is a powerful pressor which regulates basal vascular tone and glomerular hemodynamics in the cardiovascular system. ET-1 is linked to the enhancement and progression of vascular and cardiac hypertrophy, as well as to a variety of kidney diseases^54^. Interestingly, there is crosstalk between ANG II and ET-1, as the former stimulates the latter's secretion, leading to a positive feedback loop^55^. ET-1 turns out or mediates several ANG II's vascular effects, and prolonged ANG II infusion has been shown to stimulate vascular and renal ET-1 production in rats^56^. Additionally, glucocorticoids have a significant impact on the synthesis of angiotensinogen in the liver by increasing its plasma level^57^. Glucocorticoids possess a considerable vasoconstrictive reaction to Ang II. This relation indicates a greater sensitivity of glucocorticoids to Ang II, and results in increasing concentrations of Ang II type 1 (AT1) receptors centrally and peripherally^58^. Additionally, Cushing's syndrome patients have higher plasma levels of ET-1 as a result of glucocorticoids increasing of ET-1 secretion^59^. Additionally, glucocorticoids inhibit the appearance of the Na^+^/Ca^2+^ exchanger in VSMCs, and thereby increase cytosolic Ca^2+^, prompting vasoconstriction. Glucocorticoids also enhance the action of catecholamines, causing sympathetic nervous system upregulation^60^. Another important molecule in the cardiovascular system is TGF-β which represents the prototype member of a broad family of modulatory proteins involved in intercellular signaling^61^. This molecule mediates renal tubulointerstitial fibrosis^62^, and regulates fibrotic reconstruction which decreases left ventricular remodeling^57^ and renal fibrosis^63^. This principle is supported by the current histopathological findings. In the ongoing investigation, Dex treatment for 6 weeks led to rises in Ang II, ET-1, and TGF-β levels. These observations were consistent with previous research indicating that Dex (2.5 mg dexamethasone/L drinking water) increased serum and hepatic renin (angiotensinogen) substrate in rats^64^. Furthermore, numerous studies corroborated the present study’s findings, as Dex triggered endothelin production in human umbilical veins, and endothelial cell cultures in VSMCs of rats and rabbits^65,66^, and when rats were given Dex (2 mg/kg/day) for 12 days^67^. Dammeier et al.^68^ made a similar TGF-β finding and demonstrated that Dex increased connective tissue growth factors and promoted fibrosis in a variety of tissues. Notably, the current study demonstrated that PRV combination treatment at all doses significantly decreased Ang II, ET-1, and TGF-β serum levels demonstrating that the apitherapy combination may modulate hypertension-related factors. These findings supported those of Sun et al.^27^ in spontaneously hypertensive rats. Moreover, propolis' antihypertensive effects have been examined in a range of animal models and have been linked to several biological activities, including vascular reactivity modulation, and anti-inflammatory and antioxidant properties^69^.

Vitamins, carbohydrates, peptides, proteins, and fatty acids are abundant in RJ^70,71^, and certain peptides in RJ inhibit the activity of the angiotensin I-converting enzyme (ACE), resulting in hypotension in spontaneously hypertensive rats^72^. OS is another possible mechanism by which glucocorticoids induce hypertension by inhibiting both nitric oxide synthase (NOS) synthesis and transmembrane l-arginine transfer, resulting in increased peripheral resistance. Although Dex is used to treat inflammatory and autoimmune disorders, Hasona et al.^73^ reported that chronic Dex use decreases the antioxidant capacity of renal tissue. As a result, reactive oxygen species (ROS) are formed. Additionally, Dex inhibits endothelium-dependent vasodilation of resistance arterioles by inhibiting endothelium nitric oxide synthetase (eNOS), a potent vasodilatory enzyme^74^. When eNOS is inhibited, vasoconstriction occurs and then leads to hypertension. OS promotes smooth muscle proliferation and monocyte/macrophage invasion, both of which lead to vascular disease, vascular tone modification, and matrix metalloproteinase activation^75^. The present study revealed that chronic Dex treatment resulted in OS through decreased SOD, CAT, and GSH concentrations. Additionally, an increase in lipid peroxidation was evidenced by an increase in serum MDA. These findings corroborated previous research which indicated that Dex was associated with increased OS^76^.

SOD is a critical endogenous enzyme which exists in several forms and functions as a first line of defense in the battle against ROS^77^. Many studies have revealed a decrease in SOD activity in hypertensive patients and OS experimental models^78,79^. The significant increase in SOD activity seen in a dose-dependent manner in this study’s PRV treated groups suggested that PRV may act as a protective compound against oxidative injury. It is concerning that MDA is used as an OS biomarker^80^. Therefore, the high SOD elevation likely indicates a rapid rate of lipid peroxidation^81^. The present study's significant increase in MDA levels in Dex-treated rats demonstrates that Dex is a potent inducer of oxidative injury. Furthermore, the significantly decreased MDA levels observed in the PRV treated groups compared with Dex-treated groups suggested that the PRV combination may play a role in lowering lipid peroxidation.

Flavonoids have received much attention from cardiovascular researchers. Flavonoids play a role in the treatment of hypertension as it has been reported that they have vasodilatory and antihypertensive effects^82^. Because of the abundance of these compounds in propolis, it can help treat a variety of cardiovascular diseases. The ability of flavonoids to scavenge free radicals is responsible for all of their therapeutic implications^83^. Propolis also contains caffeic acid and benzyl caffeate, which inhibit the formation of lipid peroxides, and its extract possesses anti-lipid peroxidative properties^83,84^. Another antioxidant biomarker is CAT, which is a peroxidase enzyme that performs a pivotal function in the metabolism of ROS and OS adaptation^85^. Glutathione is an important intracellular thiol-disulfide redox buffer. GSH has a sulfhydryl group that is easily oxidizable and thus protects against oxidant injury^86^. The capacity of propolis to modify cardiovascular disease indicators is assumed to be due to its antioxidant features. Teles et al.^87^ reported that propolis' antioxidant and anti-inflammatory capabilities were able to prevent hypertension and structural kidney damage in Wistar rat models. Additionally, Salmas et al.^88^ reported that propolis might protect chronic hypertensive rat kidney tissue from oxidative damage. Furthermore, according to Maqsoudlou et al.^89^, because it scavenges ROS, RJ has a significant antioxidant action. Additionally, RJ has a direct relaxing effect on VSMCs by relaxing the endothelium bared aortic rings^90^, and has been shown to increase MDA and SOD concentrations in diabetic patients' erythrocytes^91^.

Bee venom (apitoxin), contains a combination of ingredients with proven therapeutic efficacy^38^. Melittin, one of the major ingredients of bee venom, stimulates the hypophyseal-adrenal system and the release of cortisone. The power of this ingredient exceeds that of hydrocortisone by 100 times as an anti-inflammatory and immunomodulator. Additionally, lysosome cell membranes are stabilized by melittin, which provides anti-inflammatory benefits. The other primary ingredients of pharmacological importance are apamin and adolapin, which block microsomal cyclooxygenase. Adolapin also inhibits thromboxane and lipoxygenase. Melittin also inhibits inflammation-related C3 of the complement system, in addition to inhibiting phospholipase A2^92^. The individual ingredients, as well as whole bee venom, protect the myocardium by regulating calcium and magnesium ions and reducing inflammation^93,94^. Bee venom has also been shown to reduce peroxidation of lipids in rats caused by lipopolysaccharide and carbon tetrachloride^95^. Furthermore, propolis and RJ have been shown to lower CAT and GSH^96^.

Hypertension is highly influenced by distinct pathophysiologic processes such as inflammation, and cardiac and vascular remodeling^97^. The nuclear factor kappa-light-chain-enhancer of activated B cells (NF-κB) modulates immune and inflammatory responses to infections by regulating pro-inflammatory gene expression^98^. The activation of NF-κB has also been linked to a variety of kidney diseases^86^. The inhibition of the NF-κB system decreases renal injury^99^. NF-κB is a significant factor in the pathogenesis of cardiovascular disorders because it regulates multiple genes, including cytokines, binding proteins, ROS, NOS, and angiotensinogen, and other products involved in atherosclerosis, immunological response, inflammation, and proliferation. There is cross talk between NF-κB and Ang II, as demonstrated by Ruiz-Ortega et al., who found that NF-κB activation occurred through AT1 and Ang II receptor type 2 (AT2), implying a role for NF-κB in cardiovascular diseases^100,101^. OS is another principal mechanism by which Ang II activates NF-κB^98^. In cardiac remodeling, long-term NF-κB activation is cytotoxic and increases heart failure by creating a persistent inflammatory response^102^. The current study demonstrated significant increases in serum NF-κB after Dex treatment. This result was explained by Queisser and Schupp^103^ who indicated that Dex stimulates mineralocorticoid receptors in cardiac and renal tissues or by activating OS. While PRV at all doses significantly reduced NF-κB, these reductions were related to the flavonoids (the main component of propolis) which act as NF-κB inhibitors. Thus, flavonoids can promote pro-inflammatory gene expressions, leading to a reduction in the inflammatory responses that underpin hypertension and some cardiovascular diseases^104^. RJ can lower NF-κB levels and have an anti-inflammatory effect^105^. Additionally, by blocking NF-κB signaling pathways, apamin, a component of bee venom, possess potent anti-inflammatory properties^42^. In terms of structural integrity of the present histopathological findings, Dex treatment triggered cardiac hypertrophy, fibrosis, and inflammatory infiltration. This observation was consistent with findings by Sun et al.^27^. The current study findings were also consistent with an earlier study that found Dex administration caused kidney injury and significant cardiac hypertrophy in rats^76^. The observed cardiac hypertrophy and remodeling resulting from Dex treatment were thought to be facilitated by stimulation of the peroxisome proliferator activator receptor α (PPAR-α), as discovered by Bernal-Mizrachi et al.^106^. Furthermore, Dex increases ACE levels and generates cardiac and renal fibrosis^107^. Prolonged Ang II elevation promotes multiple signaling cascades via AT1 receptor activation, leading to fibrosis and hypertrophy^107^. Fibrosis is characterized by the deposition of collagen fibers in the interstitial spaces which contribute to left ventricular cardiac hypertrophy^46^.

In the present study, histopathological analysis revealed that PRV treatment reversed myocardial damage and fibrosis associated with Dex-induced hypertension in a dose-dependent manner. Similarly, Wang et al.^108^ claimed that propolis treatment for 4 weeks reduced cardiac fibrosis in myocardial infarction rat models. One of the criteria for effective antihypertensive therapy is the ability to lower blood pressure while also reducing fibrosis and hypertrophy associated with myocardial remodeling, as seen in the present results using a PRV combination treatment.

Several human studies^109,110^ have observed a bidirectional relationship between arterial stiffness and hypertension. Arterial remodeling is a powerful mediator in the correlation between blood pressure variability and the incidence of cardiovascular diseases^110^. Likewise, hypertension has been associated with adventitial collagen deposition and stiffness in the aorta, as well as a reduction in arterial elasticity in mice treated with Ang II^109^. Dex-HT has been shown to cause autonomic imbalance, increase arterial stiffness, decrease baroreflex gain, and to generate cardiac remodeling^110^. Thus, Dex increased aortic fibrosis in the current investigation on one hand, but on the other hand, PRV improved aortic remodeling and decreased fibrosis in a dose-dependent manner at all doses. Similarly, Ge et al.^111^ found that losartan decreased fibrosis.

One of the primary causes of chronic kidney disease is hypertension^112^. Renal failure is a significant leading cause for cardiovascular diseases, such as hypertension, peripheral vascular disease, coronary disease, and heart failure^113^. Furthermore, hypertension nephropathy is associated with renal fibrosis, which is a prominent pathogenic characteristic. The severity of interstitial fibrosis is linked to the course of chronic renal disease^114^. According to Zhong et al.^115^, inflammation and cellular infiltration are two more important features of hypertensive renal disease. In the present study, Dex-HT resulted in kidney fibrosis, glomerular degeneration, and tubulointerstitial inflammatory infiltrations. PRV combination at all doses significantly improved the kidney histopathologically. The present apitherapy combination decreased renal fibrosis and inflammatory infiltration in a dose-dependent manner and restored an almost normal pattern. Furthermore, renal fibrosis and myofibroblast proliferation have been linked to higher TGF-β levels^116^, which is also consistent with present findings.

Because several apitherapy products possess therapeutic applications, some authors have advocated for combining individual apitherapies to achieve superior effects as cardiovascular protectants^117,118^. It has been suggested that the effects evidenced in the results are related to the phytocomplex of the mixture of bee products. Thus, more research is needed to ascertain whether the observed benefits are related to synergistic effects or to individual chemicals. Further investigation is also required to compare the impacts of apitherapy combinations with individual apitherapy products. To be more conclusive, more research, either in vitro or in clinical trials, is required.

Standardization is necessary for long-term testing to be accomplished, so to accomplish this, it is important to test for bee product allergies, especially bee venom and RJ. The dosages of bee products must be precisely calculated and adjusted over time in accordance with patient age, weight, and health status, as well as application frequency. To reach the affected and targeted areas, various administration routes should be examined. Depending on the patient's circadian rhythm and prognosis, the course of treatment may vary. Every patient must be treated individually because they are all unique, especially when it comes to treating chronic disorders which requires persistence^19^. Bee products are thought to work together, especially when accompanied by a healthy lifestyle. Additionally, not everyone responds to treatments in the same manner, so a progressive approach is advised^119^.

The quality of the products used in apitherapy is subject to factors such as soil, climate, methods of harvesting and storage, and botanical sources. Thus, it is important to establish the conditions in which the products are obtained in order to prevent patient exposure to risks of contamination and to improve results^22^.

Although many people believe that natural remedies are risk-free, it remains to be seen if there are any detrimental repercussions or interactions between bee products and medications that are widely used to treat various diseases. We again stress the importance of improving communication among beekeepers, apitherapists, researchers, nutritionists, doctors, sellers, and users of bee products.

## Conclusion

In conclusion, PRV, particularly at a higher dose of 2 mg/kg, had antioxidant, anti-inflammatory, and cardioprotective effects. Treatment of Dex-induced hypertensive rats with a PRV formula reduced hypertension and alleviated Dex's arrhythmogenic effect. These beneficial therapeutic effects could be credited to increased antioxidant activity and to the reduction of hypertension and remodeling biomarkers such as Ang II, ET-1, and TGF-β*.* PRV appeared to exhibit anti-inflammatory effects via decreasing NF-κB, and by reducing both lipid peroxidation and OS indices. Furthermore, the PRV combination significantly improved cardiac and vascular remodeling and reversed hypertensive nephropathy. These findings suggest that PRV combination therapy could be used for clinical applications in a variety of cardiovascular diseases and could effectively replace synthetic pharmaceuticals. More study is needed to establish the potency, tolerability, exact dosages, and purity needed to achieve the desired health outcomes. Additionally, RJ and bee venom are phytocomplex compounds, and the synergy of the action of the various components of propolis, RJ, and bee venom focus attention not on a single compound but on the properties of its phytocomplexes. The requirement for standardization of the processes of collection, identification, and manufacturing of formulations is based on the specific bee products, and may be achieved by evaluating absorption, distribution, metabolism, excretion, and toxicity properties of the bioactive ingredients. The delivery route must also be considered to ensure accurate results. It is crucial to identify the marker components thought to be involved in the operating mechanisms which in this work appear to be flavonoids. To assess their biological characteristics and therapeutic potential, apitherapists and researchers should investigate similar samples and determine the effectiveness of various bee products relative to one another.

Although expertise related to bee products has improved significantly, we still need to increase awareness and share this information for the benefit of humanity, which is now easier in the internet age.

## Materials and methods

### Animals

This study was conducted at the Department of Pharmacology, Faculty of Medicine for Girls, Al-Azhar University, Cairo, Egypt. Thirty-six adult albino male Wistar rats (weighing 180–240 g) were purchased from AL Nile Animal Farm, Cairo, Egypt. The animals were given one week to acclimatize to their surroundings. The rats were kept in a well-controlled environment with a 12 h light/dark cycle. The rodents were housed at a reasonable and constant temperature [23–27 Celsius (C)] and relative humidity (52–58%) and had access to their standard pellet chow during the day. The rats were habituated to their feeding regimen a week ahead of the experiment. All experimental protocols were approved by the Research Ethics Committee of Al-Azhar University's Faculty of Medicine for Girls (No. 202060306) and were implemented in accordance with the Guide for the Care and Use of Laboratory Animals of the National Institutes of Health (NIH Publication No. 85–23, revised 1996).

### Formulation of apitherapy

The PRV mixture was prepared with propolis, RJ, and bee venom. Worker bees (*Apis mellifera* L.) collected crude propolis from poplar trees, imported from Tongrentang Group Co., Ltd., Beijing, China as dust formulation (50%) and dissolved in 100% ethanol, then ultra-sonicated (GA92-II DA Ultrasonic cell grinder, China) at the power of 100 W at 40 kHz for the duration of 30 min. After that, propolis was extracted for 6 h at 45 °C and to ensure the efficiency of the extraction procedure, propolis remained submerged in alcohol for 1 day at room temperature (30 °C) using ethanol 80% with a solid to solvent ratio of 1:100. This ultrasonic-assisted extraction method was completed as per Cao and Yang ^120^. The extract was filtered by vacuum filtration and then concentrated at 60 °C. Following two further extractions of the deposit, the extract was purified and concentrated at 45 °C. After removing the wax, the ethanol-extracted propolis was dried under vacuum at 4 °C to a constant weight.

The total flavonoid content was 487.16 ± 2.33 mg, calculated as the catechin equivalent/g dry weight of the extract. A colorimetric assay utilizing aluminum chloride [Sigma-Aldrich (St. Louis, MO)] was used to assess the total flavonoid content ^121^. First, 0.3 mL of 5% sodium nitrite and 1 mL of the sample (10 mg/mL) were combined, and they were put into the reaction mixture. Five minutes later, we added 0.3 mL of 10% aluminum chloride. After another 6 min, we added 2 mL of 1 M sodium hydroxide (NaOH), directly followed by 2.4 mL of distilled water, yielding a final volume of 10 mL. at 510 nm. Finally, we assessed the flavonoid-aluminum complex's color intensity. The calibration curve (Y = 1.04597 X + 0.004523; r 2 = 0.98713) was created using catechin standard solutions (0.022–1.5 mM), which served as the internal standard, and corresponded to the absorbances (y) of the reference solutions for dosage of total flavonoids (the amount represented by x). R2 is the correlation coefficient factor used in Pearson's correlation test.

During this procedure, the following factors were observed: selectivity, linearity, precision, accuracy, detection limits, and quantification limitations.

To guarantee the accuracy of the findings, validation was carried out in accordance with the National Institute of Metrology, Standardization, and Industrial Quality (INMETRO) methodologies^122,123^. The analyses were conducted in triplicate.

May throughout June, bee venom was collected using an electronic collector from hybrid bees (A. mellifera & Carnica logistica L.) and maintained at the Plant Protection Department Apiary at the Faculty of Agriculture, Al-Azhar University, Nasr City, Cairo, Egypt (QF-1 type; Qingsong Electronic Appliance Co., Ltd., Fuzhou, Korea). Impurities were eliminated after 15 min of centrifugation at 4000×*g* at 40 °C with crude bee venom that was mixed in a 1:1 ratio mixture of 100% ethanol and deionized water. The supernatant was then filtered with a 0.45 µm membrane, the solvent was extracted in vacuum at 45 °C and the dry bee venom was kept at 20 °C until use. The composition ratio of the formulation is 100:100:0.1 (ethanol-extracted propolis: RJ: bee venom, mass ratio). A 1:1 mixture of 100% ethanol and distilled water was used to dissolve the compound, which was fully soluble in the mixture.

### Experimental design

#### Induction of hypertension

The herein experiment ran for six weeks, where the rats were randomly separated into two groups: a control group (n = 6) and a hypertensive group (n = 30). Additionally, the hypertensive group was divided into 5 smaller ones: Model, PRV 0.5 mg/kg, PRV 1 mg/kg, PRV 2 mg/kg, and losartan groups as depicted in Table 5. The control group was given saline (1 mL/kg) once a day whereas the hypertensive groups were given Dex. via a subcutaneous (SC) injection (10 μg kg day^−1^) in a volume of (1 mL/kg) to induce hypertension ^124^ at 12:00 pm for 4 weeks in the model group. As for the four remaining groups, they only received a Dex. injection for the first two weeks, then in combination with apitherapy (PRV) formula in various doses (0.5, 1.0, or 2 mg PRV/kg BW oral gavage) according to Sun et al. 2018^27^, or with losartan (10 μg kg day^−1^ oral gavage) ^125^ (Cozaar 50 mg, tablets, Merck Sharp & Dohme, UK), for the last 4 weeks of the experiment. We purchased ampoules of Dex. (each contained an 8 mg/2 mL injectable vial from Pharm. Inco. Amryia, Cairo, Egypt).

**Table 5:** The treatment of experimental animal groups.

Group	Drug	Dose	Number/group
Control group	Saline	1 mL/100 g body weight sc	6
Model	Dexamethasone	10 μg kg day^−1^	6
PRV 0.5 mg/kg	Dexamethasone + (propolis + royal jelly + bee venom)	10 μg kg day^−1^ sc + 0.5 g/kg/day oral	6
PRV 1 mg/kg	Dexamethasone + (propolis + royal jelly + bee venom)	10 μg kg day^−1^ sc + 1 g kg/day oral	6
PRV 2 mg/kg	Dexamethasone + (propolis + royal jelly + bee venom)	10 μg kg day^−1^ sc + 2 g/kg /day oral	6
Losartan	Dexamethasone + losartan	10 μg kg day^−1^ sc + 10 μg kg day^−1^ oral	6

*PRV* propolis, royal jelly & bee venom, *sc* subcutaneously.

In the present study, we examined the oral administration of the PRV mixture. To determine its efficacy, this mixture could be administered using other routes (such as injection). Further pharmacological studies are needed to better understand the pharmacokinetics of the compound, its adverse effects, and interactions that are influenced by the administration route.

#### Arterial blood pressure (ABP) & electrocardiogram (ECG) recording

##### Animal anesthesia

The rats were starved for 24 h and anesthetized with Urethane (U 2500), obtained from Sigma-Aldrich, Germany as powder. A 0.65 g/mL solution of Urethane was prepared using sterile distilled water and administered at a 1.3 g/kg in a ~ 1.5 g/5 mL solution dosage ^126^.

##### Procedure

Both ABP and ECG were recorded and analyzed using Lab Chart 8 software. An initial ECG was recorded at the end of the trial period (6 weeks). Continuous ECG data was recorded for 3.5 h using ECG electrodes, which were inserted in the anesthetized rodents’ right forearm and both right and left paws (position II). After thoroughly cleaning and shaving the ventral side of the rats' necks, right hind legs, and chests, a tiny incision (1.5–2.0 cm) was made in their necks for tracheostomy surgery and carotid artery cannulation. Then, the ABP recording was initiated. The carotid artery was located, and the blood vessel's cardiac end was occluded using a bulldog clamp for cannulation. A cannula was loaded with heparinized normal saline (0.5 IU/mL) and was used to cannulate the carotid artery. After cannulation, we slowly removed the bulldog clamp at the cardiac end of the blood vessel ^127^ then, linked the sensor to the Power Lab 4/35 hardware with Lab Chart Pro software (Animal Bio Amp Model: FE136, AD Instruments, New South Wales, Australia) and recorded the ABP data for approximately 3.5 h. According to our preliminary investigation, this period is needed to ensure the rats’ hemodynamic stability, for better recording.

#### Blood sampling and organ collection

Blood was drawn from the carotid artery cannula, used in ABP measurement. These samples were stored in test tubes and allowed to clot for 20 min at RT. The sera were then separated using a Beckman Model T-6 chilled centrifuge at 3000×*g* for 15 min, and collected in brand-new, thoroughly clean tubes. Sera were kept at −20 °C until the biochemical parameters assay. The rats were then euthanized by an overdose of anesthesia. The heart, aorta, and kidneys were removed, rinsed in cold saline, and processed for histological study.

#### Biochemical studies

Angiotensin II (Ang II) levels were assessed using an Ang II rat enzyme-linked immunosorbent assays (ELISA) kit (BG, Shanghai, China, E02A0204). ELISA kit of Endothelin-1(ET-1) was brought in from Karmania Pars GeneCo, Kerman, Iran. Transforming growth factor beta (TGF-β) was determined using rat ELISA assay kits (Thermo-Fischer Scientific Inc./Lab Vision (Fremont, CA, USA). Serum malonaldehyde (MDA), Superoxide dismutase (SOD), Catalase (CAT), and reduced glutathione (GSH) standard ELISA kits were obtained from Biodiagnostic (Giza, Egypt). To examine the NF-κB activity, a colorimetric assay was utilized using a 96-well-ELISA (Glory Science Co., Ltd, TX, USA). This assay has been shown to provide quantification of NF-κB activity in several studies. The absorbency of all examined samples was monitored using a spectrophotometric microplate reader set at 450 nm. The concentrations of all studied parameters were calculated with the standard curves.

#### Histopathological studies

The heart, aorta, and kidney specimens were fixed in a 10% formalin saline solution. After dehydration in different grades of ethyl alcohol (100%, 5 min; 95%, 2 min; 80%, 2 min; 70%, 2 min), cleaning in xylol, impregnation, and proper fixing, we embedded the specimens in paraffin wax (5 mm thick pieces). Then, we cut and fixed the specimens on glass slides using a rotatory microtome. Hematoxylin and eosin (H/E) solution (G1120, Solarbio, China) for 30 min at 55 °C were used to explore the heart's general histological structure, aorta, and kidney sections under a light microscope. The degree of cardiac fibrosis was determined using Masson's trichrome staining. Normal myocardium was stained red, while the region of cardiac fibrosis was stained blue. Slides were digitized with a 200× and 400× objective using an Olympus digital camera (Olympus, Tokyo, Japan) fitted on an Olympus microscope (Japan) with a 1/2× power adaptor.

### Statistical analysis

The normality of numerical data was investigated by examining the distribution of the data and employing normality tests (Kolmogorov–Smirnov and Shapiro–Wilk tests). The data is presented as mean ± standard deviation (SD), and range values. To determine statistical differences, a one-way analysis of variance (ANOVA) was utilized, followed by a Tukey's post hoc test. *p ˂ *0.05 as the level of significance. We used the statistical package SPSS for Windows (v 23.0; SPSS, IBM Corporation, Armonk, NY, USA).

### Ethics declarations

All experimental protocols in this study involving animals were conducted in accordance with the ethical standards and the international regulations regarding the usage and welfare of laboratory animals and were approved by the Research Ethics Committee of Al-Azhar University's Faculty of Medicine for Girls (No. 202060306), and carried out in compliance with the ARRIVE Guidelines.

## Supplementary Information

Supplementary Figure S1.

## Data Availability

The datasets used and/or analyzed during the current study available from the corresponding author on reasonable request.
