# Supplementary figures and images for: Apitherapy combination improvement of blood pressure, cardiovascular protection, and antioxidant and anti-inflammatory responses in dexamethasone model hypertensive rats
Source: Sci Rep. 2022 Dec 1;12:20765. doi: 10.1038/s41598-022-24727-z (PMC9714403; doi:10.1038/s41598-022-24727-z)

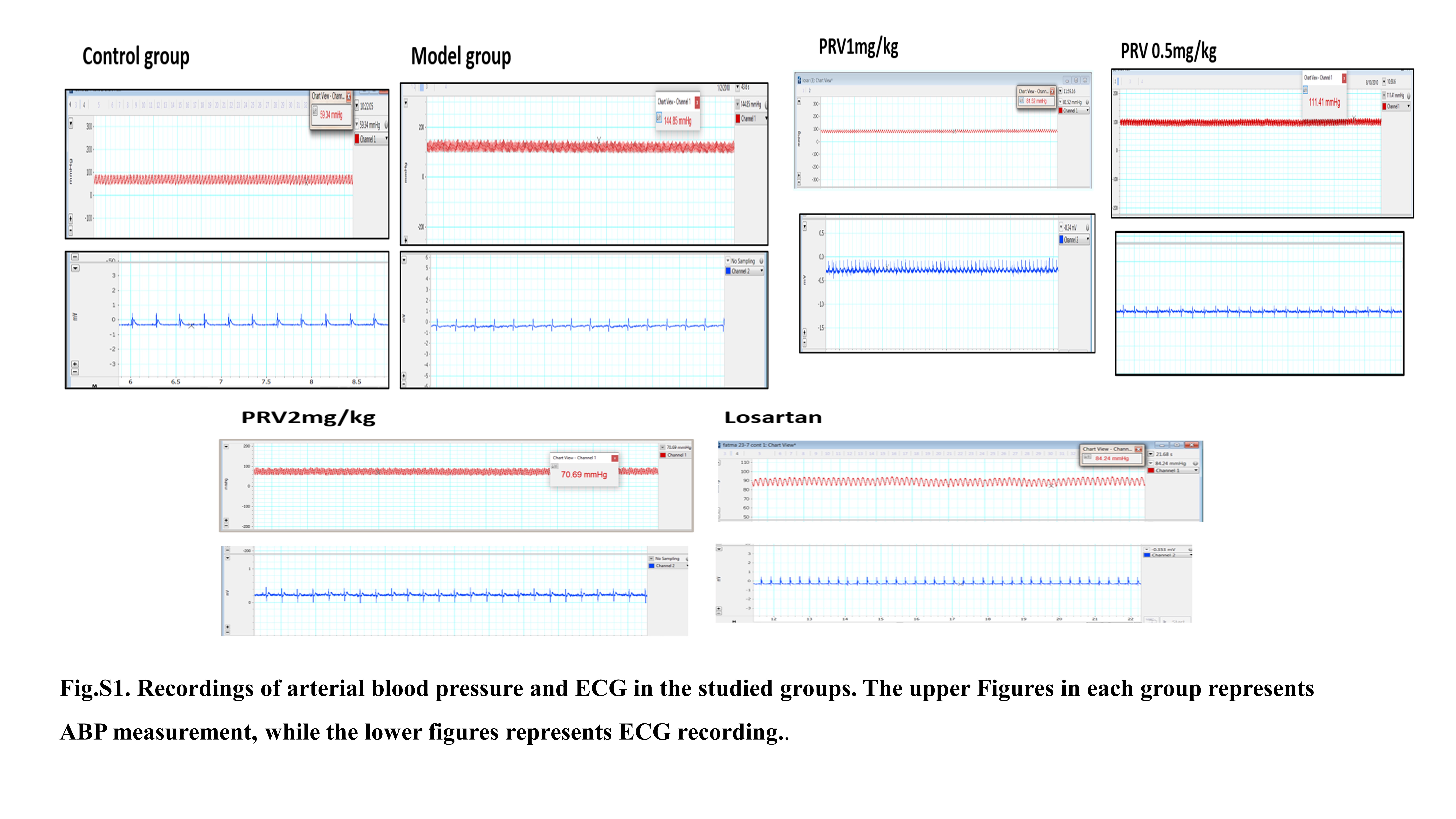

Supplement: Supplementary file 1 — Supplementary Figure S1. [file 41598_2022_24727_MOESM1_ESM.jpg]
